# Goody two plasmids: An optimized transient transfection system for AAV vector production

**DOI:** 10.1016/j.omtm.2023.06.010

**Published:** 2023-07-13

**Authors:** Sergio David Moreno Velasquez, Emma Gerstmann, Dirk Grimm

**Affiliations:** 1 Department of Infectious Diseases/Virology, Section Viral Vector Technologies, Medical Faculty, University of Heidelberg, BioQuant, Center for Integrative Infectious Diseases (CIID), 69120 Heidelberg, Germany; 2 German Center for Infection Research (DZIF) and German Center for Cardiovascular Research (DZHK), Partner Site Heidelberg, 69120 Heidelberg, Germany

The use of recombinant adeno-associated virus (rAAV) as a DNA delivery vector for human gene therapy continues to flourish, exemplified by the approval of several AAV-based gene therapy products and most recently culminating in the FDA approval of Elevidys, i.e., an AAV-mediated gene therapy for Duchenne muscular dystrophy. This rapid pace in the release of new gene therapies is unprecedented, considering that other therapeutic modalities often take around 15 years for approval,^1^ and it highlights the enormous promise and prospects of AAV vectors. Alas, the significant costs required to manufacture rAAV particles at sufficient quantity and acceptable quality continue to limit their broader application and, thus, patient access to these promising products. This, in turn, creates a profound and paramount need for affordable, scalable, and concurrently flexible innovations in AAV manufacturing to become able to meet all demands.

In a recent issue of *Molecular Therapy: Methods & Clinical Development*, van Lieshout and colleagues^2^ report a pivotal and encouraging step in this direction with their description of an optimized dual plasmid system called pOXB. It relies on the co-transfection of adherent mammalian cells with one plasmid encoding a transgene as well as the AAV genes *rep* and *cap* (plus accessory genes *AAP* and *MAAP*), together with a second plasmid providing adenoviral helper functions. Compared to other two-plasmid arrays including a mimic of the original pDG system,^3^ the pOXB configuration gave higher rAAV titers and better product quality across several rAAV genomes and AAV capsid variants, including an increased proportion of full capsids. Notably, these benefits were preserved at different manufacturing scales up to 50 L bioreactors, yielding impressive crude lysate titers of >1 × 10^15^ vector genomes per liter.

Recent experience with rAAV vectors in clinical trials has repeatedly highlighted the need for high vector doses of greater than 1 × 10^13^ particles per kg body weight when administered systemically, e.g., in patients with neurological or muscular diseases. The ensuing demand for manufacturing pipelines capable of achieving such yields in a labor-, time- and cost-effective manner is exacerbated by the wide and constantly increasing collection of clinically pertinent AAV capsid variants. These can originate from the discovery of “new” wild-type isolates or from the rapidly expanding portfolio of techniques for the derivation of synthetic capsid variants, such as directed molecular evolution, ancestral reconstruction, or machine learning.^4^ Consequently, AAV manufacturing pipelines also need to preserve a high degree of flexibility permitting the rapid accommodation of new capsid variants as they arise, yet especially those protocols based on, e.g.*,* stable producer cell lines typically requiring an early process lock.

**Figure 1:**
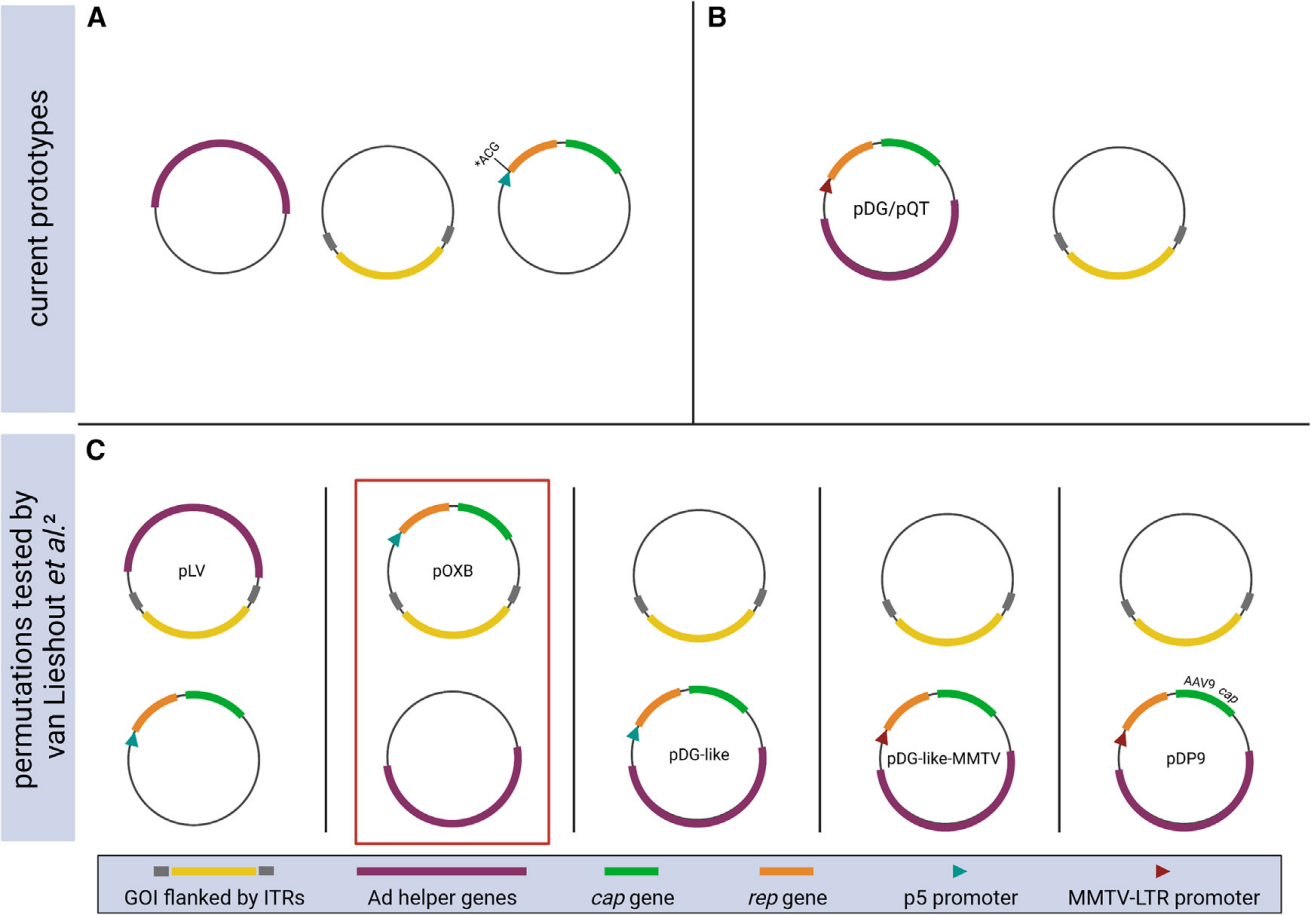
Overview over different transient transfection systems for AAV vector manufacturing Current systems are based on three (A) or two (B and C) plasmids. ∗ACG denotes a mutation of the ATG start codon for Rep78/68 that diminishes the expression of these proteins and concurrently boosts expression of the AAV capsid proteins. GOI, gene of interest; ITRs, inverted terminal repeats.

A solution that combines the best of all worlds could be provided by transient plasmid transfection systems, in which all components required for rAAV production are temporarily introduced into producer cells (typically HEK293(T)) grown in adherent or suspension cultures (Figure 1). The first systems developed in the early days of AAV manufacturing utilized one plasmid per AAV component, i.e., a rAAV plasmid encoding a gene of interest and a packaging plasmid encoding the AAV *rep* and *cap* genes.^5^ Cells that were transiently co-transfected with these two plasmids were then additionally infected with an unrelated wild-type virus, typically the name-giving adenovirus (Ad), to provide helper functions for rAAV production. As this invariably generated rAAV stocks that were heavily contaminated with a pathogenic virus, several groups quickly set out to identify the minimal set of adenoviral helper genes that suffices to support AAV production without any longer generating replication-competent Ad. Indeed, once the helper virus was replaced by a plasmid containing a mini-Ad genome encoding only E2A, E4, and VA RNA, triple transfection of the two AAV plasmids (see above) with the adenoviral helper plasmid into HEK293(T) cells that constitutively express the adenoviral E1A and E1B genes became a new and effective standard (Figure 1A).^6^^,^^7^

Since then, several major improvements of this transient transfection protocol have been reported that share the concept to combine two of the three principal components—AAV vector, AAV helper, and adenoviral helper—on one plasmid, thereby reducing the number of constructs to be transfected to two (Figures 1B and 1C). The first of these dual-plasmid systems was reported in 1998 by Grimm et al.^3^ who combined AAV *rep*/*cap* and Ad helper genes in the single helper plasmid pDG, whereas the rAAV vector was transfected *in trans*. This dual system resulted in the production of rAAV free of adenovirus and at high titer, the latter owing to the replacement of the AAV p5 promoter by a weak MMTV-LTR promoter that reduced *rep* expression and concurrently boosted expression of capsid proteins. More recently, modifications in the backbone of this archetype, most notably the replacement of the ampicillin with a kanamycin resistance gene, resulted in another dual helper plasmid called pQT that also mediates robust virus production but is more compatible with good manufacturing practices.^8^ Both systems, pDG and pQT, have in common that they are highly versatile and can readily be adapted to alternative AAV capsid variants, by simply swapping the *cap* gene within an otherwise constant plasmid backbone.^9^ Together, these earlier reports exemplified the great potential to advance transient transfection schemes through plasmid re-engineering, in turn minimizing the number of components required for AAV manufacturing and concomitantly enhancing vector yields.

In their latest work and inspired by these prior reports, van Lieshout et al.^2^ have comprehensively explored the power of dual plasmid configurations, by comparing two systems consisting of (1) a single construct containing transgene and *rep*/*cap*, while adenoviral helper functions are provided separately (pOXB), or (2) a single plasmid carrying transgene and adenoviral helper functions, while a *rep*/*cap* construct is supplied *in trans* (pLV) (Figure 1C). When these new dual systems were benchmarked against the conventional triple-transfection protocol or a pDG-like plasmid, in particular pOXB produced the highest amounts of vector genomes (VGs) at the best performing plasmid ratio. Notably, not only were the titers improved, but pOXB also generated a higher proportion of full, i.e., DNA-containing vectors (20%) compared to triple transfection (15%). Intriguingly, despite earlier evidence suggesting that downregulating *rep* results in an increased expression of capsid proteins and subsequently higher virus titers,^3^ the authors of the latest work found no difference in VG production when the native p5 promoter was replaced by the MMTV-LTR promoter, as in the broadly used pDG system.

Another consideration is the large plasmid size of pOXB, raising concerns it might be a limiting factor that could impair transfection efficiency and cell viability. Fortunately, using GFP as a transgene, flow cytometry quantification of GFP-positive cells revealed that the large size of pOXB did not impact transfection rates and thus productivity.

These observations were further extended to seven different vectors varying in size (2.2–4.6 kilobases in self-complementary or single-stranded AAV backbones, respectively), transgene, and promoter, as well as to multiple distinct capsids including AAV2 in a 2 L bioreactor context, demonstrating compatibility of pOXB with multiple AAV vectors, capsids, and scales.

To further characterize the quality of AAV vectors manufactured using pOXB, products were subjected to affinity and anion exchange chromatography, respectively. For a subset of the tested vector types, this revealed an improved proportion of intact genomes (up to 29%) concurrent with a reduction of partially packaged (up to 17%) or empty capsids (up to 13%). Importantly, particle purity, aggregation, and residual host protein contamination remained similar regardless of transfection method. Notably, despite the presence of *rep*/*cap* and the transgene flanked by the AAV inverted terminal repeats in the same plasmid, the proximity of these sequences had no detectable impact on the unwanted generation of replication-competent AAV particles. In the future, it will be informative to also study the potential emergence of capsids containing fragments of the bacterial antibiotic resistance gene or the replication origin. Further encouraging is that, using one of the vectors as example, the system could be scaled to a 50 L bioreactor, where it yielded an almost 2-fold increase in titer and proportion of full vectors compared to the triple-transfection protocol.

Finally, the authors used pOXB to produce a rAAV containing a phenylalanine hydroxylase (PAH) gene, which was then systemically injected into *Pah*^enu2^ mice. Serum samples showed phenylalanine levels over time that were similar to those obtained with vectors produced by standard triple transfection, demonstrating robust and dose-dependent *in vivo* performance of pOXB-derived vectors comparable to established AAV manufacturing pipelines.

Transient transfection of plasmids is one of the most common platforms for rAAV generation due to its many primary benefits, i.e., speed, ease, and flexibility. Furthermore, despite frequent questions about the scalability of the approach,^10^ data by the authors of this latest work^2^ as well as by others clearly show that transient transfection is indeed a viable strategy for commercial supply of AAV vectors that can be scaled to at least 2000 L bioreactors. In the future, and with the aim to further optimize and streamline transient plasmid transfection protocols, it will be highly informative to better dissect and understand the mechanisms underlying the benefits of the pOXB system. Curiously, while pOXB produced more overall capsids compared to the triple-transfection standard and akin to all other dual-plasmid systems tested in parallel, it yielded the highest titers of genome-containing particles and the lowest percentage of empty capsids. This effect was not related to the use of the AAV p5 or the MMTV-LTR promoter for *rep* expression, as experimentally ruled out by the authors, but it may be due to an ideal stoichiometry of transgene, *rep*/*cap*, and adenoviral functions that is only provided by pOXB of all tested systems. Moreover, it will be helpful and imperative to assess and ideally validate the benefits of the pOXB design with additional AAV capsid and genome variants and to benchmark its performance and product quality against other, non-transfection-based protocols such as stable producer cells or baculovirus-based manufacturing systems. Until then, the development of the innovative pOXB dual-plasmid system and its initial characterization, implying high productivity combined with great flexibility and improvements in product quality, already illustrate the vast potential of AAV/Ad plasmid re-engineering and represent a seminal cornerstone of future AAV manufacturing.
